# Primary carcinoid tumors of the liver

**DOI:** 10.1186/1477-7819-6-91

**Published:** 2008-08-27

**Authors:** Gary Schwartz, Agnes Colanta, Harold Gaetz, John Olichney, Fadi Attiyeh

**Affiliations:** 1 Department of Surgery, 1000 10th Avenue, Suite 2B, New York, NY, 10019, USA; 2 Department of Pathology, St. Luke's-Roosevelt Hospital Center, 1000 10th Avenue, 1st Floor, New York, NY, 10019, USA; 3 Department of Hematology-Oncology, St. Luke's-Roosevelt Hospital Center, 350 West 58th Street, New York, NY, 10019, USA

## Abstract

**Background:**

Primary carcinoid tumors of the liver are uncommon and rarely symptomatic. The diagnosis of primary hepatic etiology requires rigorous workup and continued surveillance to exclude a missed primary.

**Case Presentation:**

We present a case of a 62-year-old female with a primary hepatic carcinoid tumor successfully resected, now with three years of disease-free follow-up. We present a review of the current literature regarding the diagnosis, pathology, management, and natural history of this disease entity.

**Conclusion:**

Primary carcinoid tumors of the liver are rare, therefore classifying their nature as primary hepatic in nature requires extensive workup and prolonged follow-up. All neuroendocrine tumors have an inherent malignant potential that must be recognized. Management remains surgical resection, with several alternative options available for non-resectable tumors and severe symptoms. The risk of recurrence of primary hepatic carcinoid tumors after resection remains unknown.

## Background

Although carcinoid tumors can be found throughout the body, 90% occur within the gastrointestinal tract [1]. They preferentially metastasize to the liver and occasionally (< 10%) cause the carcinoid syndrome by secretion of serotonin and its precursors, as well as other vasoactive substances [2]. Primary carcinoid tumors of the liver are exceedingly rare, with only about 60 cases reported in the current literature. Meticulous follow-up is necessary to rule out an occult extrahepatic malignancy with hepatic metastasis to confirm the primary nature of hepatic carcinoids.

## Case presentation

EG is a 62-year-old female who presented with right upper quadrant abdominal pain, intermittent in timing and dull in nature, not related to oral intake and not associated with nausea or vomiting. Her past medical history included hypertension, irritable bowel syndrome, osteoarthritis, and a history of recurrent bilateral lower extremity deep venous thrombosis on Warfarin. On physical exam there were no abdominal scars, normal bowel sounds on auscultation, minimal right upper quadrant tenderness to palpation, no rebound tenderness or guarding, no hepatomegaly and a negative Murphy's sign. Her laboratory studies were significant for a GGT of 162 U/L (normal 5–80 U/L), with otherwise normal liver function tests. Tumor markers were negative, with an AFP of 3.1 ng/ml.

**Figure 1:**
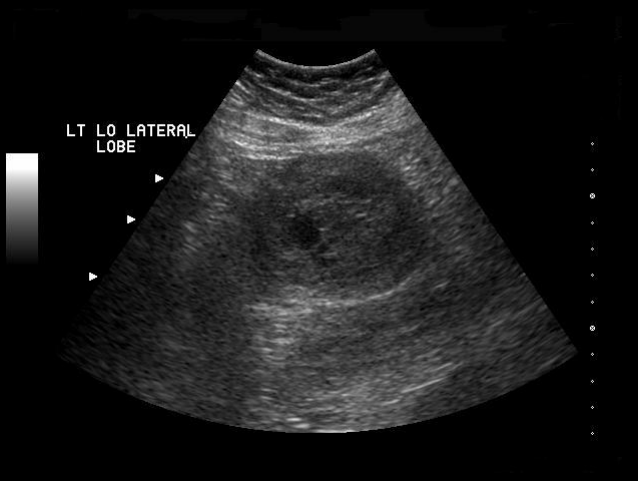
**Ultrasound of the abdomen; Ultrasound of the abdomen depicting a 6.3 × 5.3 × 5.0 heterogenous solid mass in the lateral segment of the left lobe of the liver**.

**Figure 2:**
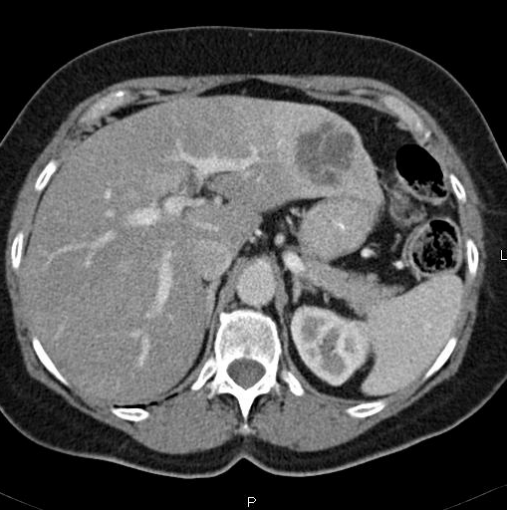
CT scan of the abdomen and pelvis; CT scan of the abdomen and pelvis with IV contrast demonstrates a 4.9 × 4.9 cm enhancing, poorly marginated mass in segment II of the liver.

Diagnostic imaging included an abdominal ultrasound (Figure 1) which revealed a heterogeneous solid mass in the lateral segment of the left hepatic lobe measuring 6.3 × 5.3 × 5.0 cm. A CT scan with intravenous contrast was obtained which revealed a 4.9 × 4.9 cm enhancing, poorly marginated mass in segment II of the liver, with no other intra-abdominal masses or lymphadenopathy (Figure 2).

A CT-guided biopsy was performed which yielded scant tissue with poorly cohesive cells arranged in papillae. PAS-D stain showed focal, small mucin droplets in some cells. Immunohistochemistry was positive for CEA and CK-7 and negative for calretinin, CDX-2, CK-20, Muc-2 and Muc-6. The limited sample was diagnosed as papillary adenocarcinoma, favoring metastasis, on the basis of morphology, special stain results and immunoprofile. However, a second panel of immunohistochemical stains for synaptophysin, CD56 and chromogranin were performed on the biopsy specimen. The tumor cells were negative for chromogranin but expressed synaptophysin and CD56, consistent with the immunoprofile of a neuroendocrine tumor (NET).

Further workup for a primary tumor or other metastatic sites included a negative CT scan of the chest, upper and lower gastrointestinal endoscopy, and a Technetium-99m bone scan. The decision was made to resect the hepatic tumor.

An uncomplicated left lateral segmentectomy (II & III) and cholecystectomy were performed. No peritoneal carcinomatosis was noted upon exploration. The postoperative course was uneventful and she was discharged home on the fourth postoperative day.

**Figure 3:**
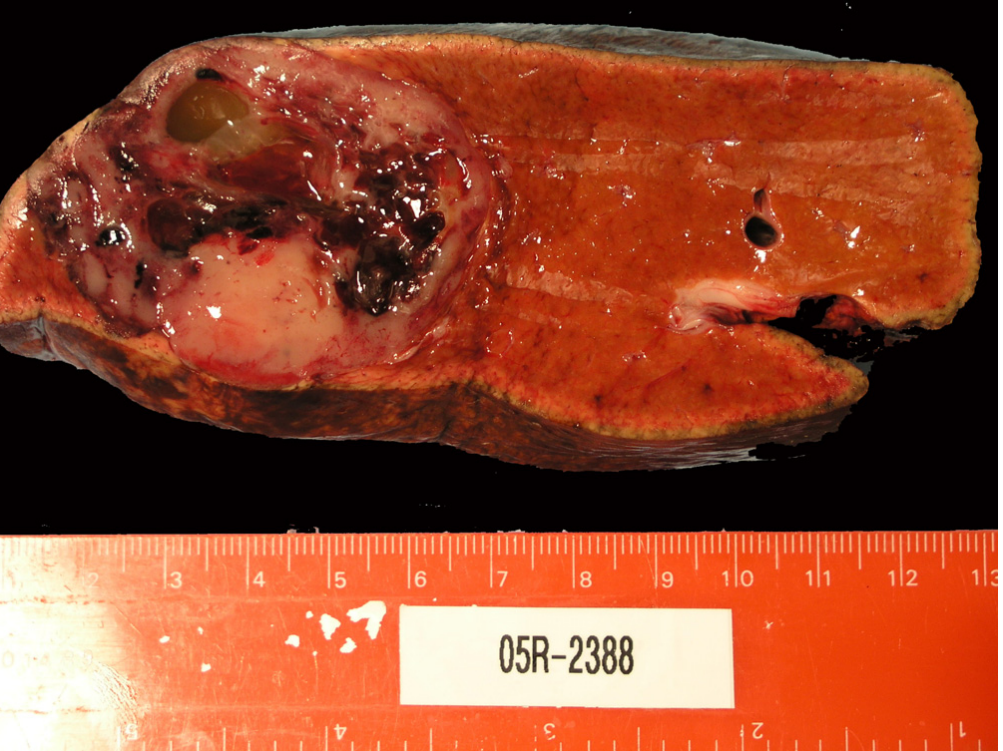
Gross image of the specimen; The specimen was measured at 5.2 × 5.0 × 5.0 cm and had a tan gray, soft, fish-fleshy cut surface.

Grossly, the tumor measured 5.2 × 5.0 × 5.0 cm and had a tan gray, soft, fish-fleshy cut surface (Figure 3). Although there was a focal infiltrative edge, it was well-circumscribed and located 5.9 cm away from the resection margin. The tumor consisted of approximately 40% solid areas and 60% hemorrhagic and cystic degenerative areas. There were no satellite nodules. Surgical margins were negative for malignancy, including the left hepatic artery, vein, duct, gallbladder and portal lymph nodes.

**Figure 4:**
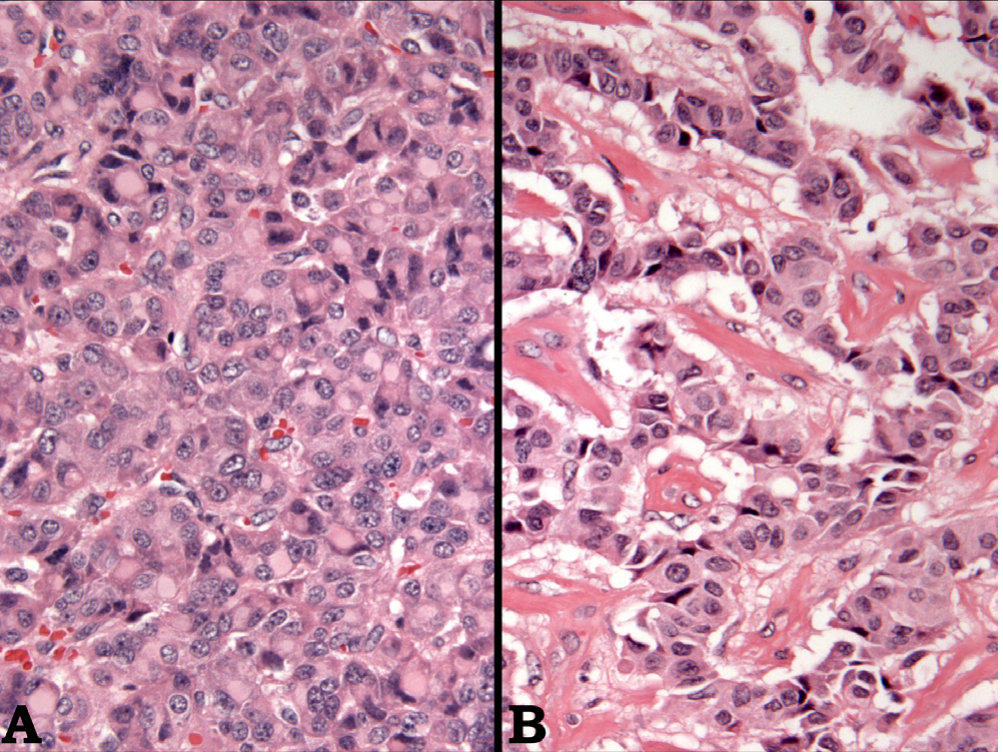
Microscopic image of the specimen; The tumor consisted of solid sheets and organoid nests of uniform, intermediate-sized, polyhedral cells in a vascular stroma (Image A) as well as areas of trabecular arrangement with focal stromal hyalinization (Image B).

Microscopically, the tumor consisted predominantly of solid sheets and organoid nests of uniform, intermediate-sized, polyhedral cells (Figure 4A) in a vascular stroma. Other areas showed a trabecular arrangement of these cells with focal stromal hyalinization (Figure 4B); cystic areas were also present. Cytologically, the tumor cells had a moderate amount of eosinophilic cytoplasm with perinuclear eosinophilic inclusions and round to oval nuclei with vesicular to finely granular chromatin. There were no areas of necrosis, and mitoses were infrequent.

**Figure 5:**
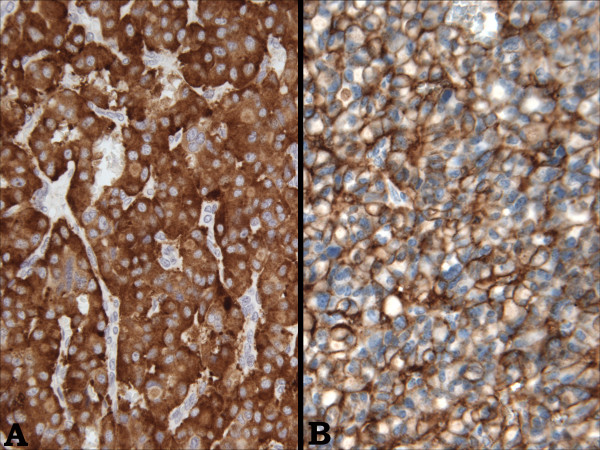
Immunohistochemistry of the resected specimen; Immunohistochemistry was positive for synaptophysin (Image A) and CD56 (Image B), consistent with a NET.

Immunohistochemistry was consistent with the immunoprofile of the biopsy specimen, i.e. positive staining for synaptophysin (Figure 5A) and CD56 (Figure 5B) and negative staining for chromogranin. Additionally, there was immunoreactivity for epithelial markers CK-7, CAM 5.2 and pancytokeratin AE1/AE3. There was negative staining for HEPT, CA19.9 and TTF-1, thus ruling out hepatocellular carcinoma, metastatic carcinoma from the gastrointestinal tract and metastatic lung carcinoma, respectively. The histomorphologic features coupled with the immunohistochemical results supported the diagnosis of a carcinoid tumor/low grade NET.

Follow-up over the subsequent three years included CT scans of the abdomen at six month intervals. To date, no recurrent or metastatic disease has been identified. She remains symptom free and in good health.

## Discussion

A total of sixty cases of primary hepatic carcinoid have been reported, with the largest series being eight patients [3], with long-term follow-up ranging from two to eleven years. Of the reported cases, there is a wide range of age at presentation and there does not seem to be gender predominance. There is no apparent association with cirrhosis or preexisting liver disease.

Primary hepatic carcinoid tumors may be an incidental finding or can present with severe symptoms including abdominal pain, jaundice, palpable right upper quadrant mass, carcinoid syndrome [4], carcinoid heart disease [5], and Cushing's Syndrome [6]. Less than 10% of gastrointestinal carcinoids present with the carcinoid syndrome and when the syndrome is present it is almost always associated with hepatic metastasis. Interestingly, the syndrome is rarely present in primary hepatic carcinoid tumors, with only two reported cases [4,5].

Imaging studies of any hepatic mass should begin with ultrasound and a triple-phase CT scan. One report supports the use of contrast-enhanced ultrasound, although there is limited experience with that modality [7]. MRI is increasingly being used, with improved visualization of carcinoid tumors on T2-weighted images [8]. Additional information can be gained from nuclear medicine imaging scans, specifically utilizing Technetium-99m isotopes, as was done with our patient [9]. Finally, if carcinoid is diagnosed postoperatively on histopathology, workup for a primary gastrointestinal site should continue with upper and lower gastrointestinal endoscopy, if these were not performed preoperatively.

The differentiation between primary and secondary NETs of the liver is not possible by histology alone, although a centrally located solitary tumor may suggest a primary [3]. Additionally, some epithelial tumors (e.g. well-differentiated hepatocellular carcinoma, adenocarcinomas and other neoplasms) may exhibit a NET-like morphology. In such cases, immunohistochemical staining for neuroendocrine markers (e.g. chromogranin, synaptophysin, CD56) should be performed to establish the cell of origin. However, it should be noted that most laboratories, including our own, use chromogranin *A *monoclonal antibody to stain for NETs, therefore NETs expressing chromogranin *B *may be non-reactive with this antibody, as was the case with our specimen.

All neuroendocrine tumors have malignant potential. As such, some authors recommend using the terms "low-grade neuroendocrine tumor," "well-differentiated neuroendocrine tumor," "well-differentiated endocrine tumor" or "grade I neuroendocrine carcinoma" instead of "carcinoid tumor" to emphasize their biologic behavior. The value of the term "neuroendocrine tumor" reflects a particular phenotype that may respond to specific targeted therapies [10].

Despite the classic low-grade cytoarchitectural morphology present in this patient's tumor, its large size (5.2 cm in greatest dimension) and focally infiltrative border are worrisome. As a general principle, NETs smaller than 1.0 cm, in any anatomic location, usually behave in an indolent fashion with only rare recurrences or distant spread while those larger than 2.0 cm are usually more aggressive [11]. However, this parameter may not be as important in primary hepatic NETs when it is noted that some of the reported cases have tumors ranging in size from 3.0–16 cm and six of eight patients have remained disease-free after follow-up of more than three years [3]. On the other hand, there are reported cases with sizes ranging from 8.2–26 cm where three of five patients died as early as seven months post-operatively [12]. The large size of the tumors in this particular series was surmised to be the cause of the unfavorable outcome.

After the appropriate workup of a hepatic mass, initial management is surgical resection when possible. Extent of resection is determined by location and size of the tumor(s), with multicentric bilobar disease often precluding resection. When this is the case, alternative therapies include radiofrequency ablation [7], hepatectomy with transplantation [3], selective hepatic artery embolization [13], regional or systemic chemotherapy, and intravenous octreotide infusion for symptomatic relief. The limited experience with this disease entity makes current recommendations of management difficult. Traditional approaches to hepatic tumors are employed at the discretion of the treating surgeons, gastroenterologists, interventional radiologists, and oncologists.

The rigorous follow-up and frequent monitoring of patients with hepatic carcinoid also serves as screening for recurrent disease. Recurrences have been reported as early as one year postoperatively and as late as thirteen years, and can occur in the liver or in regional lymph nodes [14-16]. Distant metastasis without primary hepatic recurrence has not been reported.

## Conclusion

Carcinoid tumors involving the liver are common, but primary hepatic carcinoid tumors are rare. Classification as a primary hepatic tumor requires extensive workup and prolonged follow-up. Regardless of their size, location, and degree of differentiation, NETs have an inherent malignant potential that must be recognized. Management remains surgical resection, with several alternative options available for non-resectable tumors and severe symptoms. The risk of recurrence of primary hepatic carcinoid tumors after resection remains unknown.

## Competing interests

The authors declare that they have no competing interests.

## Authors' contributions

GS drafted the case presentation and literature review sections of this manuscript. AC and HG reviewed the specimens and drafted the review of the pathological findings associated with this disease entity. FA and JO were the primary physicians diagnosing, treating, and currently following the referenced patient.
